# Duplication of the Sphenomandibular Ligament

**DOI:** 10.7759/cureus.1783

**Published:** 2017-10-18

**Authors:** Emily Simonds, Joe Iwanaga, Rod J Oskouian, R. Shane Tubbs

**Affiliations:** 1 Seattle Science Foundation; 2 Swedish Neuroscience Institute; 3 Neurosurgery, Seattle Science Foundation

**Keywords:** anatomy, cadaver, ligament, temporal bone, sphenoid bone, variation, anomaly, anesthesia

## Abstract

The normal origin of attachment of the sphenomandibular ligament is from the spine of the sphenoid bone and derailment of its course might interfere with mandibular nerve anesthetic blockade. During routine dissection of the skull base and mandibular region, a case of an anatomical variation of the sphenomandibular ligament was observed. The ligament was found to be composed of two parts; an anterior part with a wide origin from the spine of the sphenoid bone and a posterior part arising from the mandibular fossa of the temporal bone. This case and related literature were reviewed. To our knowledge, a split sphenomandibular ligament has not been previously reported. Such a variation should be kept in mind by oral surgeons and dentists during procedures in this area such as inferior alveolar nerve anesthetic blockade

## Introduction

The sphenomandibular ligament (SML) develops from Meckel's cartilage and is flat and thin [1]. The superior attachment site of the ligament is the spine of the sphenoid bone [2] and the inferior attachment is on and around the lingula of the mandible. However, variations of the SML have been reported. For example, Garg and Townsend discussed ligaments ranging in shape from short to broad and bi-concave [3]. In all cases, the ligament was surrounded by fascia and could be found in the pterygomandibular space. Some have also described the SML as a continuation of fibers entering either the petrotympanic or squamotympanic fissures to attach onto the malleus of the middle ear [4].

The function of the SML has been reported to prevent inferior distraction of the mandible [5] as when the temporomandibular joint (TMJ) is in a closed position, the SML is slack. However, the most important relationship of the SML is its relationship to the inferior alveolar nerve and nerve to the mylohyoid [4].

Herein, we report an unusual origin of the SML and discuss this in regards to other salient reports from the literature.

## Case presentation

**Figure 1:**
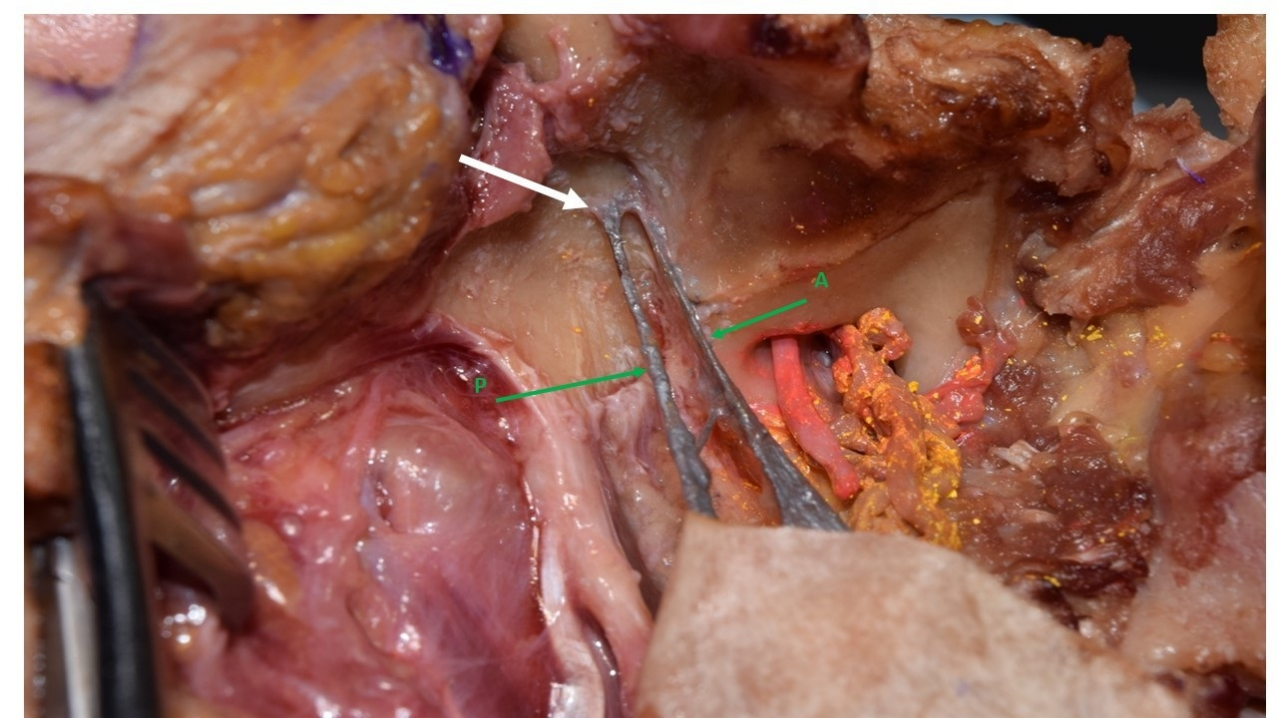
Lateral view of the right SML (gray) The upper part of the ramus of the mandible has been removed. Note the origin of the posterior SML (white arrow) was the temporal bone. A) anterior SML; P) posterior SML SML: sphenomandibular ligament

The head of a fresh frozen cadaveric 91-year-old Caucasian female was dissected. During dissection, an unusual variant of the SML was identified. The SML in this specimen was found to have a dual origin and thus appeared to be duplicated. The origins were from the sphenoid and temporal bones. One part had a wide origin from the spine of the sphenoid bone and extended to the petrotympanic fissure (anterior SML). The spine of the sphenoid bone was not well-developed on this side. The other part originated from the mandibular fossa of the temporal bone (posterior SML) (Figure 1).

**Figure 2:**
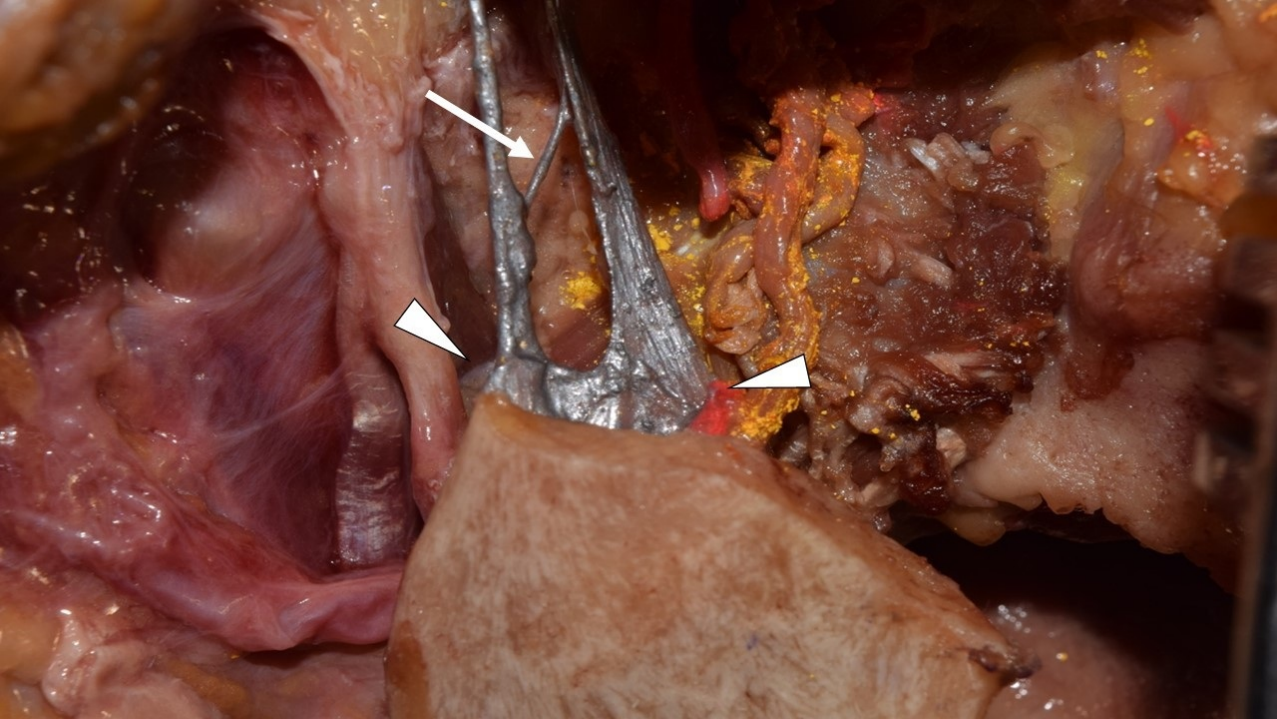
Lateral view of the attachment of the SML Note the anterior and posterior SML have a common attachment (arrowheads) with a small communicating fiber (arrow). SML: sphenomandibular ligament

Both SMLs shared a common wide distal attachment onto the lingula of the mandible. The two ligaments also had a communicating band between them near their insertion (Figure 2).

## Discussion

It is important to note that the variation in the site of attachment of the SML is significant embryologically. The SML has three distinct regions. Embryologically, the anterior and posterior parts of Meckel’s cartilage undergo ossification that later contributes to the malleus, incus, and mandible. The middle region does not ossify and becomes the SML [2].

The spine of the sphenoid bone is known as the origin of the SML. According to Burch, 88.2% (45/51) of SMLs originated from the petrotympanic fissure and 11.8% (6/51) originated from the spine of the sphenoid bone [4]. Ouchi investigated 98 sides from Japanese cadaveric heads and found that 20.4% (20/98) of ligaments originated from the spine of the sphenoid bone (type I), 34.7% (34/98) from the spine and petrotympanic fissure (type II), and 44.9% (44/98) from the spine, petrotympanic fissure, and retrodiscal tissues (type III) [6]. The spine of the sphenoid bone was the most developed in type I ligaments, and this author speculated that this was due to tension from the sphenomandibular ligament [6].

The present case demonstrated a posterior SML arising from the mandibular fossa of the temporal bone and an anterior SML arising from the spine of the sphenoid and petrotympanic fissure. This wide attachment seems similar Ouchi’s type III classification, although the origin of this SML in our case had two heads. Interestingly, the older term for the SML was the tympanomandibular ligament or malleolomandibular ligament [2, 7] as the ligament might have continuity with the malleus of the middle ear. In the present case, however, the posterior part of the SML was only attached to the surface of the mandibular fossa of the temporal bone. Such an irregular ligament might impact an inferior alveolar nerve blockade [8]. The SML variation identified here may act as a barrier to an injection of the blockade in a case where the injection is given too shallow.

## Conclusions

It is important for dentists, oral surgeons, and maxillofacial surgeons to note that such a variation of the SML, as reported herein, might exist. How such a variant affects mandibular function remains to be determined. A variation like this could complicate an inferior alveolar nerve blockade. We believe that our case is unique and, to our knowledge, has not been previously reported in the extant literature.
